# Temperature Modulation of a Catalytic Gas Sensor

**DOI:** 10.3390/s141120372

**Published:** 2014-10-29

**Authors:** Eike Brauns, Eva Morsbach, Sebastian Kunz, Marcus Baeumer, Walter Lang

**Affiliations:** 1 Institute for Microsensors, -Actuators and -Systems (IMSAS), University of Bremen, Otto-Hahn-Allee NW1, Bremen 28359, Germany; E-Mail: ebrauns@imsas.uni-bremen.de; 2 Institute of Applied and Physical Chemistry (IAPC), University of Bremen, Leobener Str. NW2, Bremen 28359, Germany; E-Mails: Morsbach@uni-bremen.de (E.M.); sebkunz@uni-bremen.de (S.K.); mbaeumer@uni-bremen.de (M.B.)

**Keywords:** catalytic gas sensor, fast response, temperature modulation, harmonics, spectrum

## Abstract

The use of catalytic gas sensors usually offers low selectivity, only based on their different sensitivities for various gases due to their different heats of reaction. Furthermore, the identification of the gas present is not possible, which leads to possible misinterpretation of the sensor signals. The use of micro-machined catalytic gas sensors offers great advantages regarding the response time, which allows advanced analysis of the sensor response. By using temperature modulation, additional information about the gas characteristics can be measured and drift effects caused by material shifting or environmental temperature changes can be avoided. In this work a miniaturized catalytic gas sensor which offers a very short response time (<150 ms) was developed. Operation with modulated temperature allows analysis of the signal spectrum with advanced information content, based on the Arrhenius approach. Therefore, a high-precise electronic device was developed, since theory shows that harmonics induced by the electronics must be avoided to generate a comprehensible signal.

## 1. Introduction

Gases are gaining increasing attention as a clean energy carrier. Notably hydrogen has a great potential in energy storage and as a carrier since it can be generated with the help of renewable energies e.g., [1]. A fast and reliable detection of gas leakages is necessary to avoid accumulation due to the resulting danger of explosion. A high number of gas sensors has been developed as a simple monitoring system solution. The most common applied approaches are based on metal oxide semiconductors (MOS) and catalytic combustion, e.g., [2–4]. The working principle of a MOS-based gas sensor is a change of conductivity of the functional metal oxide layer caused by reducing or oxidizing gases [3,4]. The working principle of a catalytic gas sensor is a change of temperature due to the exothermic combustion of the detected gas [3,4]. All principles have run through a number of developments, predominantly based on miniaturization, e.g., to reduce power consumption and response time. To achieve small time constants and high thermal isolation, thermopiles on thin membranes made of silicon nitride are usually used [5].

However, major challenges in catalytic gas sensing are still their selectivity and signal stability. While the signal intensity of different gases differs, identification of individual gases is not possible. A gas that causes a low sensor signal at high concentration can be confused with a gas that causes a high sensor signal at low concentration, since selectivity is just an issue of different reactivity. To determine the real gas concentration, the gas present has to be known. Furthermore, the sensor characteristics change over time due to drift effects, influencing the signal stability or perhaps influenced by the environmental temperature.

A new catalytic gas sensor was developed with a focus on small dimensions and small amounts of catalytic material to create a fast sensor device [6–9]. A catalytic combustion-based sensor was chosen, since that principle offers high dynamics due to a low response time, a low recovery time and no need of an additional recovery modus as in case of most MOS-based sensors [2–4,8]. Using this sensor design enables measuring advanced gas characteristics by modulation of the catalyst temperature and application of the Arrhenius approach as shown in [10,11]. A closer look at the behavior, theoretical background and technical requirements will be presented in this work.

## 2. Technical Implementation

### 2.1. Device

**Figure 1.**
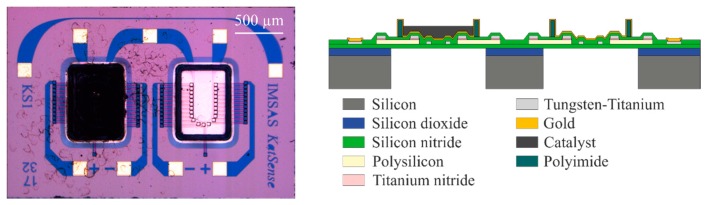
Microscope picture of the sensor (**left**) and a scheme of the sensor structure and materials (**right**).

Figure 1 shows the sensor, as well as a scheme of the sensor structure. It consists of a membrane with catalyst and a reference membrane, both made of low stress silicon-rich silicon nitride. The membranes are prepared by low pressure chemical vapor deposition (LPCVD) and exhibit a thickness of 600 nm. The use of a reference module enabled us to diminish the effects that originate from fluctuation of the environmental temperature when a sensor is kept at constant temperature [7–9]. The implementation of two separate membranes minimizes the thermal interaction between catalyst and reference. A heater is integrated in both membranes to heat the catalyst and the reference membrane to the desired operating temperature. The temperature is measured by thermopiles made out of p-doped polysilicon and tungsten-titanium, with every thermopile consisting of several elements, exhibiting a high Seebeck coefficient. In order to protect the electrical structures from environmental influences, the functional layers are embedded and passivated in the membrane material. By applying LPCVD with high temperatures a pinhole free membrane with a high density is produced, which is very stable and chemically inert. To endure high LPCVD-process temperatures the heater and the thermopiles are made of tungsten-titanium. A diffusion barrier made of titanium nitride is used to avoid diffusion effects at the junctions of the thermopiles [12].

**Figure 2.**
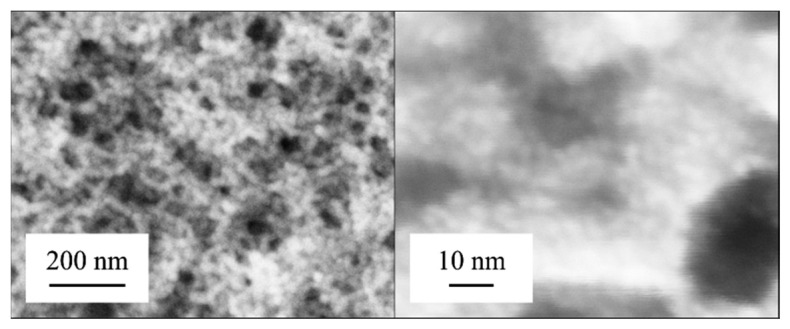
SEM pictures of platinum nanoparticles stabilized by organic ligands. The pictures illustrate the high porosity and surface. On the hand right picture, particles can be seen. Average particle size is 1.8 nm (with permission from [8]).

The catalyst is prepared on the membrane using platinum nanoparticle dispersions. To increase the stability, the nanoparticles were stabilized by organic ligands. Thereby, only small amounts are necessary to archive a high surface with a suitable high reactivity [7–9] (Figure 2). A low thermal capacity supports a low response time. In order to avoid undesired coating of other parts of the sensor, a boundary made of polyimide is generated at the edge of the membrane.

### 2.2. Electronics

**Figure 3.**
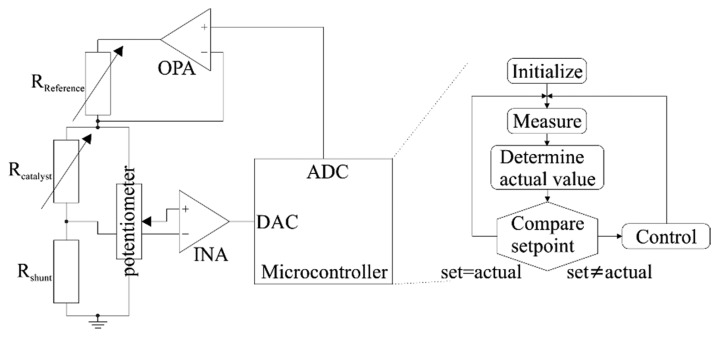
Schematic of the electronic circuit for digital controlling of the heater temperature by a microcontroller.

The temperature dependence of resistance for thin film structures is utilized to control the temperature of the heater. The heater resistance is measured by a digital circuit, which controls the power supply. The heater is contacted at three points, allowing for a selective temperature control of catalyst and reference membrane (see Figure 3). The entire heater is supplied with the same power, whereas the temperature is measured at the catalyst only. Both membranes have the same temperature as long as no combustion occurs. If the catalyst is exposed to a catalytically reactive gas, combustion by reaction with oxygen occurs. Thereby the heat of reaction is released into the catalytic layer, reducing the power demand to keep the catalyst at a constant temperature. As less power is applied to keep the catalyst at operation conditions the temperature of the reference membrane decreases. The temperature at the reference thereby correlates with the electrical power consumption. Under constant temperature control, this mode of heat control exhibits the advantage of eliminating fluctuations of the environmental temperature by establishing the difference between the catalyst and reference thermopile as the sensor signal. With modulated temperature control the electrical power signal (see Section 3.1) can be extracted directly from the sensor with the advantage of a higher resolution, and a broader detection range [13].

In Figure 3, the functionality of the electronics is shown schematically. The use of a microcontroller for time variant temperature control has the advantage of a high resolution. Compared to the use of a digital potentiometer, as shown in [10,11], the occurrence of harmonics within the basic signal can be avoided. These basic harmonics complicate the analysis of the measured data, as explained in Section 3.

## 3. Theoretical Background and Measurement Implementation

### 3.1. Arrhenius Approach

**Figure 4.**
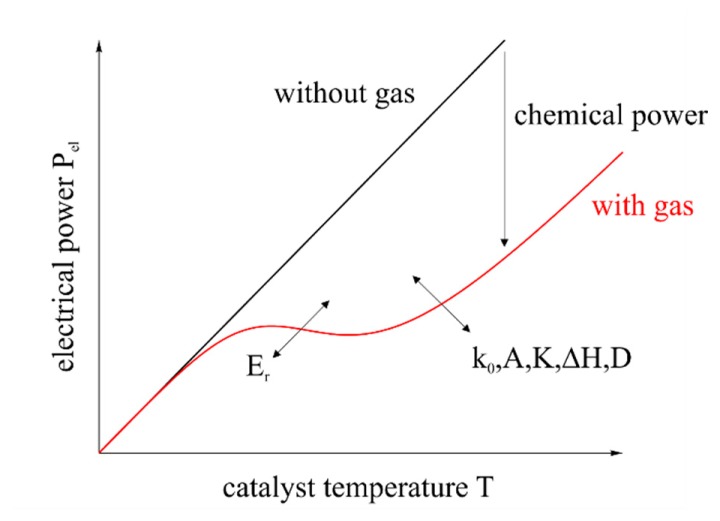
Schematic of the electrical power related to the catalyst temperature. The scheme shows a white range curve. Real measurements maximal run until point of inflection, since further behavior shows high temperatures. Technical implementation is not possible due to various reasons, e.g., catalyst stability.

The power needed to keep the catalyst at a constant temperature is given by Equation (1):

(1) $$P_{el}\left(K\right)=P_{el}\left(0\right)-{\text{P}}_{\text{chem}}\left(K\right)$$

where *P_el_*(*K*) is the electrical power consumption at the gas concentration *K*, *P_el_*(0) is the electrical power consumption without gas exposure (dependent on the environmental temperature) and P*_chem_*(*K*) is the chemical power, induced by the gas concentration *K*. The electrical power consumption is linearly related to the heater temperature as long as no catalytically convertible gas is present. The chemical power induced nonlinearity is given by the following equation [14–18]:

(2) $${\text{P}}_{\text{chem}}=\Delta H\cdot K\cdot A\frac{k\cdot \beta }{k+\beta }$$

where Δ*H* is the enthalpy of reaction and *A* is the catalytically active surface of the catalyst. *k* is the rate constant of reaction and β is the mass transfer coefficient, given by the following equations [14–18]:

(3) $$k=k_{0}\cdot e^{\frac{-E}{R\cdot T}}$$

(4) $$\beta =\frac{D}{L}$$

where *k*_0_ is the pre-exponential factor, *E* is the catalytic activation energy, *R* is the universal gas constant and *T* is the temperature. *D* is the diffusion coefficient of the gas at the catalyst and *L* is the characteristic length of the catalyst. The influence of the heat of reaction on the electrical power is shown in Figure 4. Furthermore, the effect of the given elements of the equations is shown. While Δ*H* is determined by the combustion of a given gas, the remaining elements are specific for the catalyst, its constitution (e.g., surface topography and porosity) and for the gas and can only be determined empirically.

This approach enables to determine gas characteristics by heating the catalyst sinusoidal and interpreting the harmonic behavior to identify a present gas. A sine is used, since this signal ideally induces no harmonics by itself. Experiments and calculations have shown that harmonics of the basic signal are dominant and demolish the consistent behavior shown in the following.

Using a time variant temperature controlling of the catalyst with the operating point *T*_0_, the amplitude *T_a_* and the frequency *f*:

(5) $$T=T_{0}+T_{\alpha }\sin\left(2\pi \cdot f\cdot t\right)$$

the harmonics appear as a integer multiple of the frequency *f*. This behavior can be illustrated by using Taylor series with Equation (1) [18]:

(6) $$P_{el}\left(T\right){|}_{T0}≅P_{el}{|}_{T0}+\frac{\partial P_{el}}{\partial T}{|}_{T0}\left(T-T_{0}\right)+\frac{1}{2}\frac{\partial^{2}P_{el}}{\partial T^{2}}{|}_{T0}{\left(T-T_{0}\right)}^{2}+\frac{1}{6}\frac{\partial^{3}P_{el}}{\partial T^{3}}{|}_{T0}{\left(T-T_{0}\right)}^{3}+\ldots$$

with the trigonometric relation [19]:

(7) $$\left(T-T_{0}\right)=T_{a}\sin\left(2\pi \cdot f\cdot t\right)$$

(8) $${\left(T-T_{0}\right)}^{2}=\frac{T_{a}^{2}}{2}\left(1-\cos\left(2\pi \cdot 2f\cdot t\right)\right)$$

(9) $${\left(T-T_{0}\right)}^{3}=\frac{T_{a}^{3}}{4}\left(3\sin\left(2\pi \cdot f\cdot t\right)-\sin\left(2\pi \cdot 3\text{f}\cdot \text{t}\right)\right)$$

**Figure 5.**
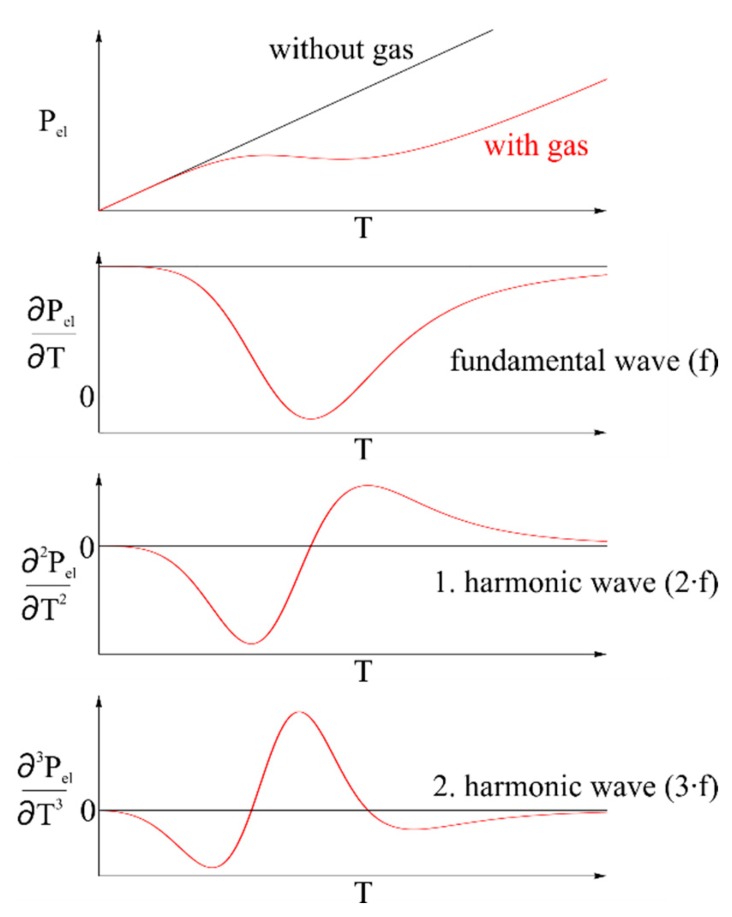
Harmonic waves of the electrical power as shown in Equation (6).

The intensity of the harmonics is shown in Figure 5.

**Figure 6.**
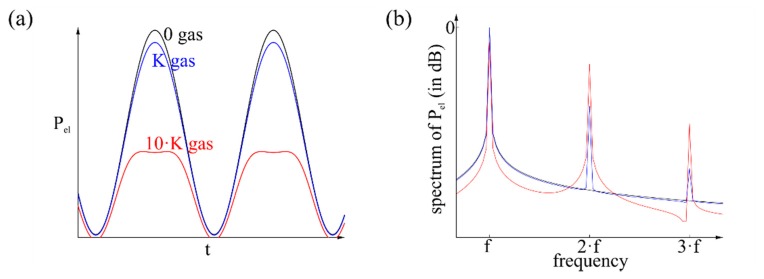
(**a**) Trend of the wave related to the time and (**b**) spectrum of the signal, both without gas, with the gas concentration *K* and with the gas concentration of 10 *K*.

A way to use the signal and to examine the gas is to analyze the spectrum of the signal as shown in Figure 6. By supplying different concentrations of the same gas the intensity of the harmonics changes, while the position on the temperature axis is the same related to Figure 5. Furthermore, different gases exhibit harmonics on different positions on the temperature axis. Related to Figure 6b, the relation of harmonics intensity is the same for the same gas with different concentrations, while the entire intensity changes. The relation changes for a different gas. This analysis is independent from drift effects or environmental temperature, because it depends on the derivation, respectively the amplitude instead of the average value. The environment-dependent term *P_el_*(0) has no more influence.

This mathematical relation only applies as long as no harmonics occur at the fundamental wave. These fundamental harmonics degrade the mathematical relation, and the influence of the gas becomes inconsistent.

### 3.2. Measurements

To avoid fundamental harmonics and to achieve a clear signal, the amplitude and the frequency have to be small, adapted to the sensors' response time and the controlling speed. Besides, high amplitudes are not necessary, since Figure 5 shows high intensity of the harmonics in a small range up to the inflection point of the electrical power. A sine with an amplitude of 5 °C (from 85 °C to 95 °C) and a frequency of 1 Hz was used.

For analysis of the sensor behavior during temperature modulation, the signal of the reference thermopile *U_TP,Ref_* was investigated, as this signal behaves proportional to *P_el_* (see Section 2). Different concentrations were created by mixing a suitable gas with synthetic air. Due to a very small test chamber and a short response time and recovery time, fast gas concentration changes can be achieved [8].

**Figure 7.**
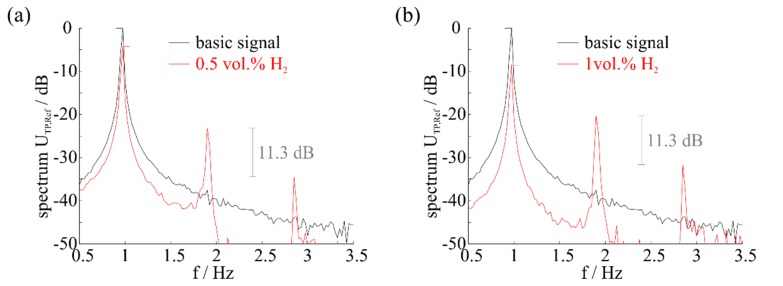
Spectrum at a gas concentration of (**a**) 0.5 vol.% H_2_ and (**b**) 1 vol.% H_2_. The intensity of harmonics differs, while the distance (relation) is 11.3 dB in both cases.

**Figure 8.**
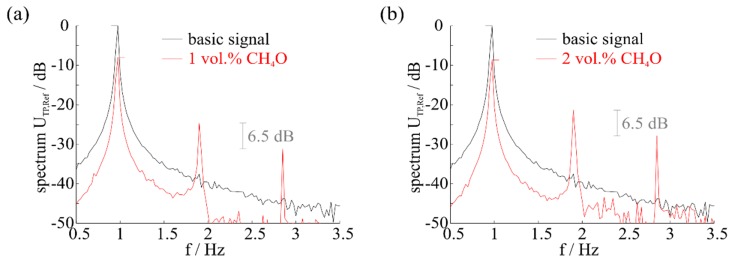
Spectrum at a gas concentration of (**a**) 1 vol.% CH_4_O and (**b**) 2 vol.% CH_4_O. The intensity of harmonics differs, while the distance (relation) is 6.5 dB in both cases.

Figures 7 and 8 show the results of measurements with hydrogen and methanol. These gases were selected, since they have the smallest selectivity in constant temperature control mode of the sensor [8], but different behavior can be observed for several combustible gases according to reproducibility.

## 4. Results and Discussion

It can be seen that a consistent behavior of the harmonics occurs as shown in the mathematical model. By determining the relation of harmonics intensity to the measurements, the spectra differ, while the relation of harmonics intensity is the same for the same exposed gas with a different concentration. If hydrogen is present in the atmosphere, the distance is approx. 11.3 dB for every concentration, while methanol offers a distance of approx. 6.5 dB.

An identification of the gas present is thereby possible by analysis of harmonics behavior or by choosing a specific temperature range that offers high harmonics for the sought gas and low harmonics for the remaining gases. However, the distinctness of the suitable concentration is complicated compared to a constant control modus [7,8], since the difference in signal is less clear, depending on the harmonics intensity. In addition a precise temperature zone needs to be investigated to achieve a good resolution. Furthermore, the potential for selective measurement and identification of the concentration depends on a very clean basic signal. Measurements as well as mathematical modelling with a basic signal including basic harmonics have shown that the relation as shown in Figures 6 and 7 will be lost, since basic harmonics are dominant. With the presence of the same gas with different concentrations the relation of the harmonics intensity is not the same.

Basic harmonics occur if the amplitude and the frequency are too high for the response time of the sensor, leading to a deformation of the sine signal. Another reason is a low resolution of the controlling circuit. Furthermore, noise occurs with the selected controlling concept if the electrical power demand is low, since a certain power is needed to create a needful feedback for the controlling loop. This happens if a low control temperature is chosen or if chemical power induction is high.

## 5. Conclusions and Outlook

The measurements and the mathematical model have shown that in principle it is possible to generate selective signals with a catalytic gas sensor by utilizing a mathematical relation of physical and chemical effects. One advantage of this method is the independence of drift effects or of any offset, caused by fluctuation of the environmental temperature, because the analysis depends on the amplitude instead of the average value.

For a better understanding of these effects and selectivity a broader study of different gases is needed. Furthermore, the electronic control of the temperature must be improved. Especially at low electrical power consumption demand which means a low control temperature or by high chemical power induction a high noise occurs. The mathematical relations as well as measurements have shown that a clean basic signal is needed to create a consistent behavior by a specific gas.

Another interesting issue is the analysis of gas mixture compositions. It may be investigated if there is no mathematical relation between the spectra of the single gases and the gas composition. Furthermore, the single gases could be identified by temperature modulation in different temperature ranges, since the position of the harmonics differ with varying gases.
